# Comparison of CO_2_ Reduction Performance with NH_3_ and H_2_O between Cu/TiO_2_ and Pd/TiO_2_

**DOI:** 10.3390/molecules26102904

**Published:** 2021-05-13

**Authors:** Akira Nishimura, Ryouga Shimada, Yoshito Sakakibara, Akira Koshio, Eric Hu

**Affiliations:** 1 Division of Mechanical Engineering, Graduate School of Engineering, Mie University, 1577 Kurimamachiya-cho, Tsu, Mie 514-8507, Japan; nisiaki531@ybb.ne.jp (R.S.); yoshitohockey@gmail.com (Y.S.); 2 Division of Chemistry for Materials, Graduate School of Engineering, Mie University, 1577 Kurimamachiya-cho, Tsu, Mie 514-8507, Japan; koshio@chem.mie-u.ac.jp; 3 School of Mechanical Engineering, the University of Adelaide, Adelaide, SA 5005, Australia; eric.hu@adelaide.edu.au

**Keywords:** CO_2_ reduction, Cu/TiO_2_ photocatalyst, Pd/TiO_2_ photocatalyst, molar ratio of reductants

## Abstract

The aim of this study is to clarify the effect of doped metal type on CO_2_ reduction characteristics of TiO_2_ with NH_3_ and H_2_O. Cu and Pd have been selected as dopants for TiO_2_. In addition, the impact of molar ratio of CO_2_ to reductants NH_3_ and H_2_O has been investigated. A TiO_2_ photocatalyst was prepared by a sol-gel and dip-coating process, and then doped with Cu or Pd fine particles by using the pulse arc plasma gun method. The prepared Cu/TiO_2_ film and Pd/TiO_2_ film were characterized by SEM, EPMA, TEM, STEM, EDX, EDS and EELS. This study also has investigated the performance of CO_2_ reduction under the illumination condition of Xe lamp with or without ultraviolet (UV) light. As a result, it is revealed that the CO_2_ reduction performance with Cu/TiO_2_ under the illumination condition of Xe lamp with UV light is the highest when the molar ratio of CO_2_/NH_3_/H_2_O = 1:1:1 while that without UV light is the highest when the molar ratio of CO_2_/NH_3_/H_2_O = 1:0.5:0.5. It is revealed that the CO_2_ reduction performance of Pd/TiO_2_ is the highest for the molar ratio of CO_2_/NH_3_/H_2_O = 1:1:1 no matter the used Xe lamp was with or without UV light. The molar quantity of CO per unit weight of photocatalyst for Cu/TiO_2_ produced under the illumination condition of Xe lamp with UV light was 10.2 μmol/g, while that for Pd/TiO_2_ was 5.5 μmol/g. Meanwhile, the molar quantity of CO per unit weight of photocatalyst for Cu/TiO_2_ produced under the illumination condition of Xe lamp without UV light was 2.5 μmol/g, while that for Pd/TiO_2_ was 3.5 μmol/g. This study has concluded that Cu/TiO_2_ is superior to Pd/TiO_2_ from the viewpoint of the molar quantity of CO per unit weight of photocatalyst as well as the quantum efficiency.

## 1. Introduction

Because of large concerns around the world, the global warming problem is a hot area of R&D. Each country has set a goal to reduce the amount of CO_2_ emissions. In Japan, the prime minister has declared the intent to reduce the effective CO_2_ emissions to zero by 2050. However, the global mean concentration of CO_2_ in atmosphere had increased up to 410 ppmV in September 2019, which was 25 ppmV increase from the value in 2009 [1]. Therefore, development of technologies which can reduce the amount of CO_2_ in the atmosphere the is urgently required.

Solar conversion of CO_2_ to fuel seems a promising procedure to solve the global warming problem for sustainable development of society. Solar energy, is the form of direct solar irradiation, is widely available and it is imperious to utilize it for solar fuel production [2]. One pathway to realize solar conversion of CO_2_ is photochemical reactions. According to a literature survey by the authors, photocatalysts can convert CO_2_ into fuel species such as CO, CH_4_, CH_3_OH, etc. [3,4,5]. TiO_2_ is a popular photocatalyst used for CO_2_ reduction since it is convenient to obtain, inexpensive, and has strong resistance to chemicals and corrosion [6]. Pure TiO_2_ can only function under UV light illumination which represent only 4% of the energy available in solar radiation [4]. CO_2_ reduction performance is thus greatly improved if the TiO_2_ or modified TiO_2_ can function under visible light illumination.

Some studies have reported on the development of various modified TiO_2_ forms [3,4,5]. The modifications included loading TiO_2_ with Au, Ag, Pd, Pt, Rh, Ir or bimetals (e.g., Ag-Au and Au-Pt) [7]. A hierarchical pore network and morphology to prepare the bio-templated TiO_2_ catalyst [8], heteroleptic iridium complex supported on graphite carbon nitride [9], TiO_2_ synthesis using superficial fluid technology [10] and N-doped reduced graphene oxide promoted nano TiO_2_ [11] were attempted modifications to prepare TiO_2_ to respond to visible light. According to a review paper on the surface modification of TiO_2_ to enhance its CO_2_ reduction performance [12], there are many approaches for the surface modification of TiO_2_ such as impurity doping, metal deposition, alkali modification, heterojunction construction and carbon-based material loading. As an example of impurity doping, the CH_3_OH production of Cu/TiO_2_ increased with an increase in the amount of Cu doping, while the over-doping of Cu would lead to high defect density in the TiO_2_, resulting in degradation of the CO_2_ reduction performance [12]. Therefore, there is the optimum ratio of dopant to enhance the CO_2_ reduction performance of photocatalyst. As to an example of heterojunction construction, it was reported that the photocatalytic CO_2_ reduction performance over Cu/TiO_2_ hollow nanoparticles was much better than that of pure TiO_2_ and Cu_2_O [12]. On the other hand, another recent review paper reported that not only Cu_2_O but also Cu_2_O/TiO_2_ hybrid photocatalyst exhibited a higher CO_2_ reduction performance compared to pure TiO_2_ [13]. Cu_2_O which has a small band gap energy (2.4 V) can help the absorption efficiency in the visible range of the solar spectrum. In addition, Cu_2_O can promote the CO_2_ reduction performance by concomitantly increasing the electron-hole separation efficiency. It was also reported that Cu_2_O/TiO_2_ proceeded the photocatalytic reaction, resulting in the increase in the selective formation of CO under the illumination condition of light whose wave length was over 305 nm.

Though various metals have been used for doping, the reductant which is a partner for CO_2_ reduction is also important. According to the literatures survey by the authros, H_2_O or H_2_ were normally used as the reductants for CO_2_ reduction [2,7]. The reaction scheme to reduce CO_2_ with H_2_O can be summarized as shown below according to the previous studies [14,15,16]:

Photocatalytic reaction

TiO_2_ + *hν* → h^+^ + e^−^ (1)

Oxidation

2H_2_O + 4h^+^ → 4H^+^ + O_2_ (2)

Reduction

CO_2_ + 2H^+^ + 2e^−^ → CO + H_2_O (3)

CO_2_ + 8H^+^ + 8e^−^ → CH_4_ + 2H_2_O (4)

The reaction scheme to reduce CO_2_ with H_2_ can be summarized as follows [17]:

Photocatalytic reaction

TiO_2_ + *hν* → h^+^ + e^−^ (5)

Oxidation

H_2_ → 2H^+^ + 2e^−^ (6)

Reduction

CO_2_ + e^−^ → CO_2_^−^ (7)

CO_2_^−^ + H^+^ + e^−^ → HCOO^−^ (8)

HCOO^−^ + H^+^ → CO + H_2_O (9)

H^+^ + e^−^ → H (10)

CO_2_ + 8H + 8e^−^ → CH_4_ + 2H_2_O (11)

In the reduction process, the same number of H^+^ and e^−^ are necessary. Since the doping metal emits the electron which is contributed to prevent the recombination of h^+^ and e^−^ [17], the number of H^+^ should be arranged. Therefore, the combination of doped metal type and reductants is important.

Though various metals have been used for doping, Cu and Pd are favorite candidates [2]. Cu can improve TiO_2_ photoactivity and selectivity in the CO_2_ photocatalytic application [2]. Cu can extend the absorption band to 400–800 nm [18,19] which covers the whole visible light range. It was reported that Cu/TiO_2_ was superior to pure TiO_2_. Cu/Cu^+^ fabricated Ti^3+^/TiO_2_ can produce 8 μmol/g of CH_4_ which is 2.6 times more than in the case of Ti^3+^/TiO_2_ [20]. Cu/TiO_2_ prepared by a facile solvothermal method had yields of CO and CH_4_ up to 4.48 μmol/g and 5.34 μmol/g, which are 10 times higher than those of TiO_2_ [21]. It was reported that the synthesized Cu_2_O/TiO_2_ showed a performance of 3.5 μmol/g of CO production while that of TiO_2_ was 0.1 μmol/g [18]. These results [18,20,21] were achieved by CO_2_ reduction with H_2_O under visible light illumination conditions. On the other hand, Pd can also extend the absorption band to 400 –800 nm [22,23], which covers the whole visible light range. Pd/TiO_2_ exhibited a higher reduction performance to produce hydrocarbons and H_2_ compared to pure TiO_2_ [22,23,24]. This is due to the work function of Pd, which reflects its electron donating or accepting ability. In addition, it is thought that Pd loaded on TiO_2_ functions to increase the efficiency of photogenerated electrons for the formation of reductive products. Pd/TiO_2_ nanowire produced 50.4 μmol/g of CO and 26.7 μmol/g of CH_4_ which were an improvement by 54% and 7%, respectively, compared to those of TiO_2_ nanowire [25]. The other study reported that the production of Pd/TiO_2_ was 3.50 μmol/g which was 2.5 times as large as that of pure TiO_2_ [26]. Pd/TiO_2_ prepared by a photochemical deposition method exhibited 0.28 μmol/g of CH_4_ which was 14 times as large as that of pure TiO_2_ (Degussa P-25) [27]. These results [25,26,27] were obtained from CO_2_ reduction with H_2_O under the visible light illumination conditions.

Though there are some reports on CO_2_ reduction with H_2_O or H_2_ [3,27], the effect of NH_3_ having 3H^+^, which is superior to H_2_O and H_2_, on CO_2_ reduction performance of photocatalyst is not investigated yet with the exception of the previous studies conducted by Nishiura et al. using Fe [28] or Cu [29]. In addition, other doped metals have not been investigated yet from the viewpoint of comparison of several metal ion types. When the combination of CO_2_/NH_3_/H_2_O is considered, the ion number of dopants is important to match the number of electrons emitted from the dopant with H^+^ as shown in the reaction scheme. The same number of electrons and H^+^ is necessary to produce fuel. The reaction scheme to reduce CO_2_ with NH_3_ can be summarized as shown below [15,30]:

Photocatalytic reaction

TiO_2_ + *hν* → h^+^ + e^−^ (12)

Oxidation

2NH_3_ → N_2_ + 3H_2_ (13)

H_2_ → 2H^+^ + 2e^−^ (14)

Reduction

H^+^ + e^−^ → H (15)

CO_2_ + e^−^ → CO_2_ (16)

CO_2_^−^ + H^+^ + e^−^ → HCOO^−^ (17)

HCOO^−^ + H^+^ → CO + H_2_O (18)

CO_2_ + 8H + 8e^−^ → CH_4_ + 2H_2_O (19)

It is thought that the total amount of electron which is needed for photochemical reaction is large due to the combination of two H^+^ suppliers such as NH_3_ and H_2_O, according to the reaction scheme. Since Pd has a high reduction performance [23,24,31] which can assist the progress of reduction reaction in CO_2_ reduction with NH_3_ and H_2_O, this study selected Pd as a dopant as well as Cu.

The purpose of this study was to clarify the effect of doped metal type on the CO_2_ reduction characteristics of TiO_2_ with NH_3_ and H_2_O. The CO_2_ reduction performance with NH_3_ and H_2_O using Cu/TiO_2_ or Pd/TiO_2_ coated on netlike glass fiber as photocatalyst has been investigated under the illumination conditions of a Xe lamp with or without UV light. In the study, the ratio of CO_2_/NH_3_/H_2_O has been set at 1:1:1, 1:0.5:1, 1:1:0.5, 1:0.5:0.5, 3:2:3, 3:8:12, respectively, to determine the optimum molar ratio of CO_2_/NH_3_/H_2_O with Cu/TiO_2_ or Pd/TiO_2_ as photocatalyst. According to the reaction scheme to reduce CO_2_ with H_2_O or NH_3_, shown above, the theoretical molar ratio of CO_2_/H_2_O to produce CO or CH_4_ should be 1:1 or 1:4, respectively, while that of CO_2_/NH_3_ to produce CO or CH_4_ should be 3:2, 3:8, respectively. Therefore, this study assumes that the molar ratio of CO_2_/NH_3_/H_2_O = 3:2:3 and 3:8:12 are theoretical molar ratios to produce CO and CH_4_, respectively.

## 2. Materials and Method

### 2.1. Preparation of Cu/TiO_2_ and Pd/TiO_2_ Photocatalyst

TiO_2_ film was prepared by sol-gel and dip-coating process [29]. [(CH_3_)_2_CHO]_4_Ti (purity of 95 wt%, Nacalai Tesque Co., Kyoto, Japan) of 0.3 mol, anhydrous C_2_H_5_OH (purity of 99.5 wt%, Nacalai Tesque Co., Kyoto, Japan) of 2.4 mol, distilled water of 0.3 mol, and HCl (purity of 35 wt%, Nacalai Tesque Co., Kyoto, Japan) of 0.07 mol were mixed for preparing TiO_2_ sol solution. This study coats TiO_2_ film on netlike glass fiber (SILIGLASS U, Nihonmuki Co., Kyoto, Japan) by a sol-gel and dip-coating process. Glass fiber having diameter of about 10 μm weaved as a net is collected to give a diameter of approximately 1 mm. The pore diameter of the glass fiber and the specific surface area are approximately 1 nm and 400 m^2^/g, respectively from the specifications of the netlike glass fiber. The netlike glass fiber is composed of SiO_2_ (96 wt%). The opening space of the net glass is approximately 2 mm × 2 mm. Since the netlike glass fiber has porous characteristics, the netlike glass fiber can capture TiO_2_ film easily during the sol-gel and dip-coating process. Additionally, we can expect that CO_2_ is more easily absorbed by the prepared photocatalyst due to the porous characteristics of the netlike glass fiber. This study cut the netlike glass fiber into disc forms having a diameter and thickness of 50 mm and 1 mm, respectively. The netlike glass disc was dipped into a TiO_2_ sol solution at the speed of 1.5 mm/s and pulled up it at the fixed speed of 0.22 mm/s. After that, the net was dried out and fired under a controlled firing temperature (*FT*) and firing duration time (*FD*) to fasten TiO_2_ film on the base material. This study set *FT* and *FD* at 623 K and 180 s, respectively.

After the coating of TiO_2_, this study loaded Cu or Pd on the TiO_2_ coated netlike glass fiber by a pulse arc plasma gun method [29] emitting nanosized Cu or Pd particles uniformly under an applied high voltage potential difference. The pulse number can control the quantity of metal loaded on TiO_2_. This study set the pulse number at 100. This study applied an ARL-300pulse arc plasma gun device (ULVAC, Inc., Chigasaki, Japan) with a Cu or Pd electrode whose diameter was 10 mm for Cu or Pd loading, respectively. After the netlike glass fiber coated with TiO_2_ was set in the evacuated vessel of the pulse arc plasma gun device, the Cu or Pd electrode emitted nanosized Cu or Pd particles by applying a voltage potential difference of 200 V. The pulse arc plasma gun can evaporate Cu or Pd electrodes into fine particle form over the target in a concentric area whose diameter is 100 mm under the condition that the distance between Cu or Pd electrode and the target is set to be 160 mm. Due to the distance between Cu or Pd electrode and TiO_2_ film of 150 mm, these conditions can uniformly spread Cu or Pd particles over the TiO_2_ film.

### 2.2. Characterization of Cu/TiO_2_ and Pd/TiO_2_ Film

This study evaluated the structure and crystallization characteristics of Cu/TiO_2_ film and Pd/TiO_2_ film by SEM (JXA-8530F, produced by JEOL Ltd., Tokyo, Japan), EPMA (JXA-8530F, produced by JEOL Ltd., Tokyo, Japan) [29], TEM (JEM-2100/HK, JEOL Ltd., Tokyo, Japan), EDX (JEM-2100F/HK, JEOL Ltd., Tokyo, Japan), STEM (JEM-ARM200F, JEOL Ltd., Tokyo, Japan), EDS (JEM-ARM200F, JEOL Ltd., Tokyo, Japan) and EELS (JEM-ARM2007 Cold, produced by JEOL Ltd., Tokyo, Japan) [32].

These measuring instruments use electrons to characterize materials, meaning that the samples should conduct electricity. Because the netlike glass disc used for base material to coat Cu/TiO_2_ or Pd/TiO_2_ film can’t conduct electricity, a carbon vapor was depositied by a dedicated device (JEE-420, produced by JEOL Ltd., Tokyo, Japan) on the netlike glass discs before characterization. The thickness of the carbon deposited on samples is controlled to be approximately 20–30 nm. The electrode emits the electrons to the sample by setting the acceleration voltage of 15 kV and the current at 3.0 × 10^−8^ A in order to analyze the external structure of samples by SEM. After the X-ray characteristics are analyzed by EPAM, the concentration of chemical elements is clarified referring to the relationship between the characteristic X-ray energy and the atomic number. SEM and EPMA have a spatial resolution of 10 mm. The EPMA analysis can help clarify the structure of the prepared photocatalysts as well as to measure the quantity of loaded metal within TiO_2_ film on the netlike glass disc as base material. The electron probe emits electrons to the sample at the acceleration voltage of 200 kV, when the inner structure of the sample is analyzed by TEM and STEM. The size, thickness and structure of loaded Cu and Pu were evaluated by TEM and STEM, respectively. The X-ray characteristics of the sample is detected by EDX and EDS at the same time, so the concentration distribution of chemical element in the thickness direction of the samples is known. The size, thickness and structure of loaded Cu and Pd were evaluated by TEM and STEM, respectively. The characterization of X-ray is detected by EDX and EDS at the same time, resulting in that the concentration distribution of chemical elements in the thickness direction of the samples is analyzed. EELS is used to detect elements as well as to determine the oxidation states of transition metals. The EELS characterization was determined by a JEM-ARM200F system equipped with GIF Quantum having 2048 ch. The dispersion of 0.5 eV/ch for the full width ad half maximum of the zero loss peak was measured in this study.

### 2.3. CO_2_ Reduction Experiment

**Figure 1:**
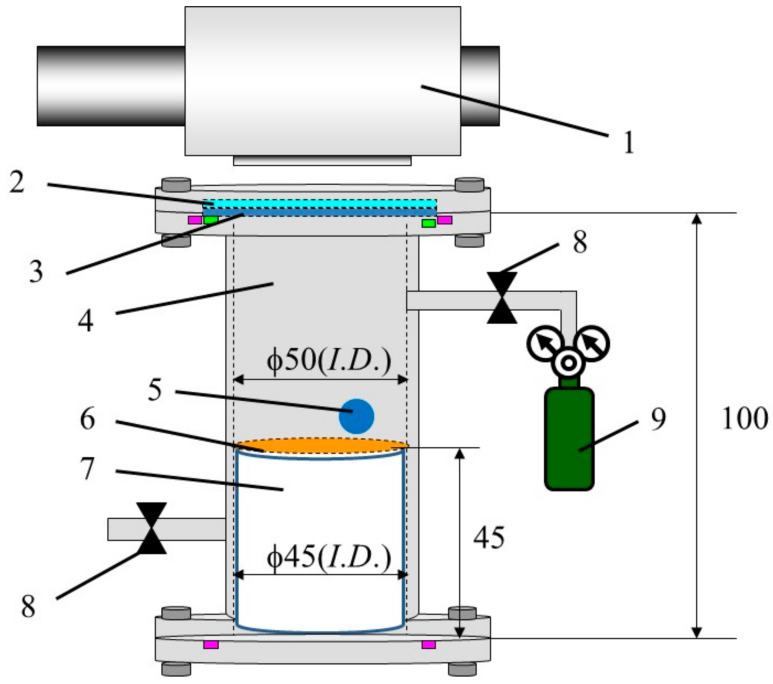
Experimental set-up for CO_2_ reduction (In this Figure, 1: Xe lamp, 2. Edge cut filter, 3. Quartz glass disc, 4. Stainless tube, 5. Gas sampling tap, 6. Photocatalyst, 7. Teflon cylinder, 8. Valve, 9. CO_2_ gas cylinder (99.995 vol%)).

Figure 1 illustrates the experimental set-up of the reactor consisting of a stainless tube with dimensions of 100 mm (*H*.) × 50 mm (*I*.*D*.), Cu/TiO_2_ film coated on netlike glass disc having the scale of 50 mm (*D*.) × 1 mm (*t*.) positioned on a Teflon cylinder having dimensions of 50 mm (*H*.) × 50 mm (*D*.), a quartz glass disc of 84 mm (*D*.) × 10 mm (*t*.), a sharp cut filter cutting off the light whose wavelength is below 400 nm (SCF-49.5C-42L, produced by Sigma Koki Co. Ltd., Tokyo, Japan), a 150 W Xe lamp (L2175, produced by Hamamatsu Photonics K. K., Hamamatsu, Japan), mass flow controller and CO_2_ gas cylinder [29]. The reactor size to charge CO_2_ is 1.25 × 10^−4^ m^3^. The light of Xe lamp which is positioned on the top of the stainless tube illuminates Cu/TiO_2_ film or Pd/TiO_2_ coated on the netlike glass disc through the sharp cut filter and the quartz glass disc that are located on the top of the stainless tube. Xe lamp has the wavelength of light ranged from 185 nm to 2000 nm. The sharp cut filter can get rid of UV from the Xe lamp, resulting that the wavelength of light illuminating to Cu/TiO_2_ film or Pd/TiO_2_ film ranges from 401 nm to 2000 nm with the filter. In this study, the average light intensity of Xe lamp without and with the sharp cut filter is 58.7 mW/cm^2^ and 47.1 mW/cm^2^, respectively.

After filling CO_2_ gas of 99.995 vol% purity in the reactor which was pre-evacuated by a vacuum pump for 15 min, the valves positioned at the inlet and the outlet of reactor were closed in the CO_2_ reduction experiment with NH_3_ + H_2_O. After that, we confirmed that the pressure and gas temperature in the reactor at 0.1 MPa and 298 K, respectively. Then, we injected NH_3_ aqueous solution (NH_3_; 50 vol%), which was changed depending on the planed molar ratio, into the reactor via gas sampling tap, and turned on Xe lamp at a time. Due to the heat of infrared light components illuminated from Xe lamp, the injected NH_3_ aqueous solution vaporized completely in the reactor. The temperature in the reactor reached at 343 K within an hour and it was maintained at approximately 343 K during the experiment. We changed the molar ratio of CO_2_/NH_3_/H_2_O at 1:1:1, 1:0.5:1, 1:1:0.5, 1:0.5:0.5, 3:2:3, 3:8:12, respectively. The reacted gas in the reactor was extracted by gas syringe via gas sampling tap and it was analyzed by FID gas chromatography (GC353B, GL Science, Tokyo, Japan) and a methanizer (MT221, GL Science, Tokyo, Japan). The FID gas chromatograph and methanizer have a minimum resolution of 1 ppmV.

## 3. Results and Discussion

### 3.1. Characterization Analysis of Cu/TiO_2_ and Pd/TiO_2_ Film

**Figure 2:**
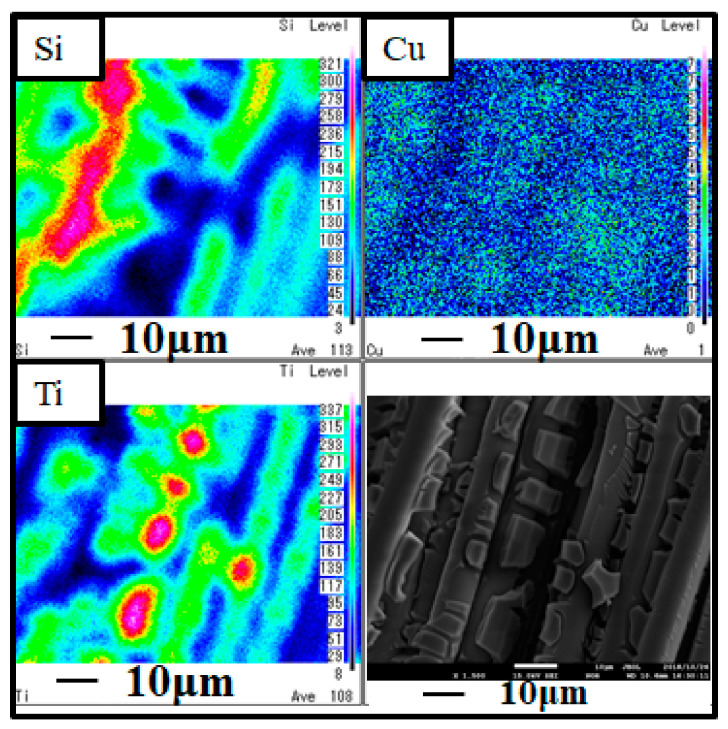
SEM and EPMA results of Cu/TiO_2_ film coated on netlike glass disc.

**Figure 3:**
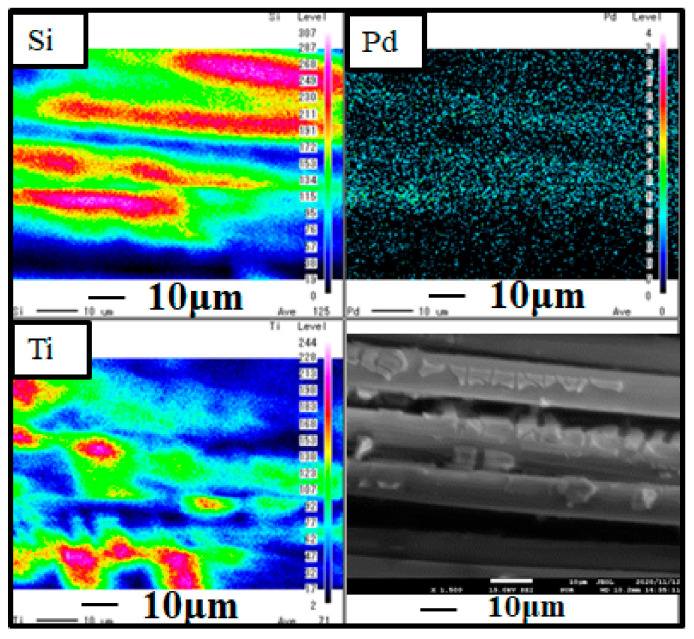
SEM and EPMA results of Pd/TiO_2_ film coated on netlike glass disc.

Figure 2 and Figure 3 show SEM and EPMA images of Cu/TiO_2_ and Pd/TiO_2_ film coated on netlike glass disc, respectively. Black and white SEM images at 1500 times magnification were obtained in this study, which were also used for EPAM analysis. As to the EPMA image, the concentrations of each element in observation area are displayed by diverse colors. Light colors, e.g., white, pink, and red are used to display a large amount of an element. On the other hand, dark colors like black and blue are used to display a small amount of element. According to Figure 2 and Figure 3, it is observed that TiO_2_ film having teeth-like shape coated on the netlike glass fiber is formed irrespective of pulse number. Since the thermal conductivity of Ti and SiO_2_ at 600 K are 19.4 W/(m·K) and 1.82 W/(m·K), respectively [33], the temperature distribution of TiO_2_ solution adhered on the net like glass disc was not even during the firing process. Thermal expansion and shrinkage around netlike glass fibers occurred, resulting in the formation of thermal cracks within the TiO_2_ film. Therefore, it is believed that TiO_2_ film on netlike glass fiber has a teeth-like form. In addition, it was found that nanosized Cu and Pd particles were loaded on TiO_2_ film uniformly. The observation area which is the center of netlike glass disc having the diameter of 300 μm was analyzed by EPMA to measure the amount of loaded Cu or Pd within the TiO_2_ film. The ratio of Cu or Pd to Ti is calculated by averaging the data detected in this area. The weight percentage of element Cu within Cu/TiO_2_ film was 1.62 wt%, while the weight percentage of element Pd within Pd/TiO_2_ film was 1.64 wt%. The weight percentages of loaded Cu and Pd were almost the same, indicating that pulse arc plasma gun method could control the amount of metal doped on TiO_2_ irrespective of metal type. On the other hand, total weights of Cu/TiO_2_ and Pd/TiO_2_ which were measured by an electron balance and averaged among 10 samples are 0.05 g and 0.07 g, respectively.

**Figure 4:**
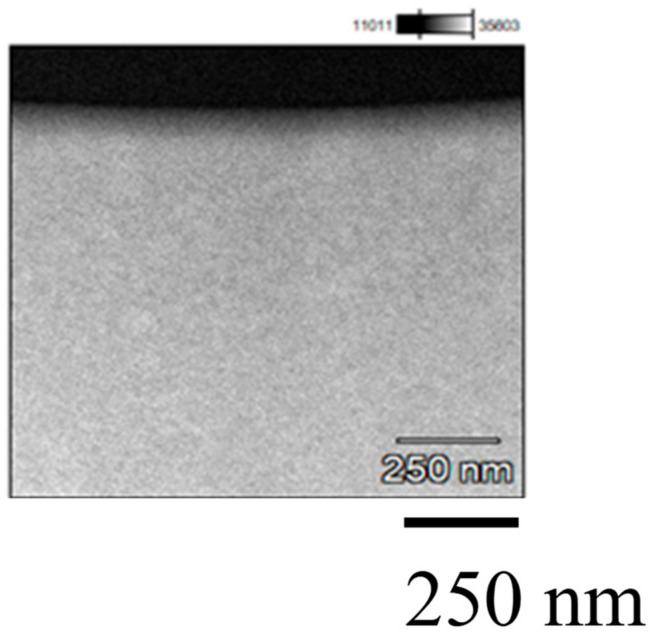
TEM images of Cu/TiO_2_ film.

**Figure 5:**
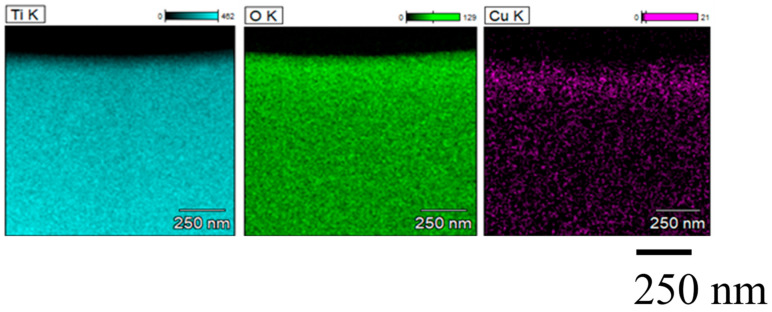
EDX images of Cu/TiO_2_ film (Left: Ti, Center: O, Right: Cu).

Figure 4 and Figure 5 show TEM and EDX images of Cu/TiO_2_ film, respectively. EDX analysis was carried out using TEM images taken at 15,000 times magnification. It is observed from Figure 5 that Cu particles are distributed in the TiO_2_ film. Although many Cu particles are loaded on the upside of TiO_2_ film, it is not confirmed that a Cu layer is formed [32].

**Figure 6:**
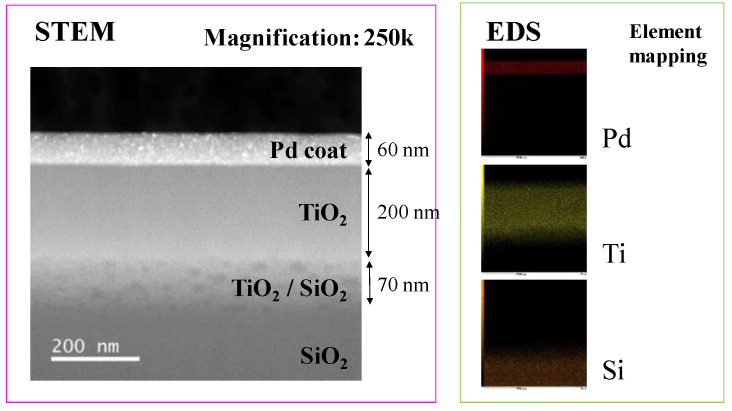
STEM and EDS result of Pd/TiO_2_ film coated on netlike glass disc.

Figure 6 shows STEM and EDS results of Pd/TiO_2_ film coated on the netlike glass disc. A 250,000 times magnification STEM image was used in the EDS. It is observed from the STEM image that Pd is coated on the TiO_2_ film, which is confirmed by EDS images, too. It is also observed that the layers of Pd and Ti are separated. It is seen that the thickness of the Pd coated is approximately 60 nm. The observation area is small compared to EPMA images shown in Figure 3, suggesting that nano-sized Pd particles are loaded on TiO_2_ dispersedly [34].

**Figure 7:**
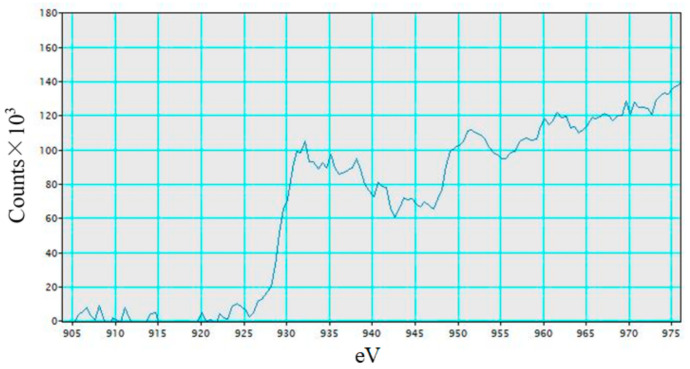
EELS spectra of Cu in Cu/TiO_2_.

Figure 7 shows the EELS spectra of Cu in Cu/TiO_2_ film. According to this figure, peaks at around 932 eV and 952 eV can be observed. Compared to a report investigating the spectral peaks of Cu_2_O and CuO [35], the EELS spectra of Cu_2_O matches Figure 7. Therefore, Cu in Cu/TiO_2_ prepared in this study exists as Cu^+^ ion in Cu_2_O. It was reported that Cu^+^ was more active than Cu^2+^ [36]. Consequently, it is expected that Cu^+^ plays a role in enhancing the CO_2_ reduction performance.

**Figure 8:**
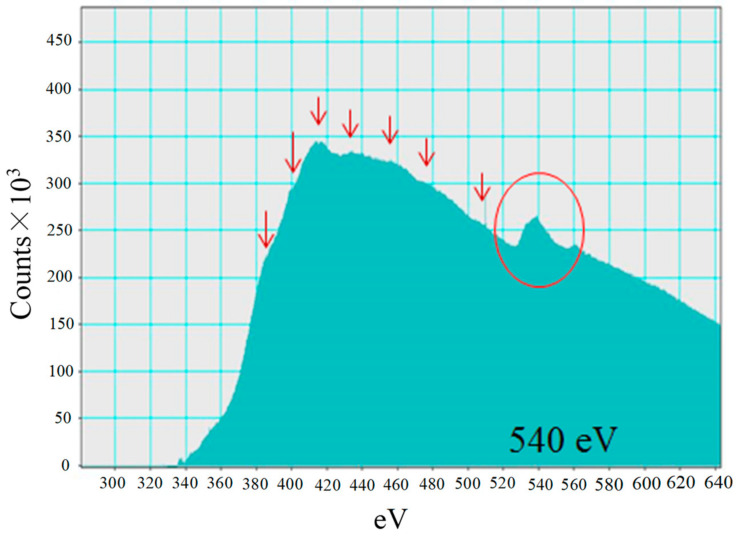
EELS spectra of Pd in Pd/TiO_2_.

Figure 8 shows EELS spectra of Pd in Pd/TiO_2_ film which displays peaks at around 540 eV. Comparing the spectra peaks of Pd nanowire with that of Pd metal and PdO [28], it is seen that the EELS spectra of Pd metal matches that in Figure 8. Therefore, it can be thought that Pd in Pd/TiO_2_ prepared in this study exists as Pd metal. Since the photoreduction performance of Pd/TiO_2_ was higher than that of PdO/TiO_2_ [31,37], it is confirmed that the desirable Pd/TiO_2_ without oxidation was prepared in this study.

### 3.2. CO_2_ Reduction Characteristics of Cu/TiO_2_

Table 1 and Table 2 list the changes in molar quantity of CO per unit weight of photocatalyst for Cu/TiO_2_ film coated on netlike glass disc with the time under the condition of Xe lamp illumination with and without UV light, respectively. In these tables, the impact of molar ratio of CO_2_, NH_3_ and H_2_O is also evaluated. In addition, fuels other than CO were not detected in this study. Before this experiment, a blank test under the condition of CO_2_/NH_3_/H_2_O or CO_2_/H_2_O without Xe lamp illumination had been carried out as a reference, resulting that no fuel was detected as expected. As to the reproducibility of experiments, this study shows the data from averaging three experiments. Table 1 and Table 2 also list the maximum value of molar quantity of CO per unit weight of photocatalyst which is written in bold font.

It can be seen from Table 1 that the CO_2_ reduction performance for the molar ratio of CO_2_/NH_3_/H_2_O = 1:1:1 is the highest where the molar quantity of CO per unit weight of photocatalyst is 10.2 μmol/g at 6 h. According to the reaction scheme of CO_2_ reduction with H_2_O or NH_3_ shown above, the theoretical molar ratio of CO_2_/H_2_O to produce CO or CH_4_ is 1:1 or 1:4, respectively. In addition, the theoretical molar ratio of CO_2_/NH_3_ to produce CO or CH_4_ is 3:2, 3:8, respectively. Based on these theoretical molar ratios, the molar ratio of CO_2_/NH_3_/H_2_O = 3:2:3 should be the theoretical molar ratio to produce CO. However, it is revealed that the molar ratio of CO_2_/NH_3_/H_2_O = 1:1:1, which exhibits the highest performance of CO production as shown in Table 1, is different from the theoretical molar ratio assumed. Since the ionized Cu doped with TiO_2_ can provide free electrons to be used for the reduction reaction process [38,39], the theoretically required quantity of the reductant NH_3_ and H_2_O is reduced from the values according to the theoretical reaction scheme with TiO_2_ i.e., CO_2_/NH_3_/H_2_O = 3:3:3 to 3:2:3. It is also observed from Table 1 that the produced CO decreases after reaching a maximum value. It is believed that the decrease in the produced CO was caused by the reoxidation reaction with CO and O_2_ [40], and not caused by the deactivation of the photocatalyst.

As to the impact of NH_3_ on CO_2_ reduction characteristics using Cu/TiO_2_, the authors’ previous study [30] had drawn the following conclusions: Comparing the concentration change of CO along the time under the Xe lamp with UV light for the molar ratio of CO_2_/H_2_O = 1:1 to that for the molar ratios of CO_2_/NH_3_/H_2_O = 1:1:1, 1:0.5:1, 3:2:3, it is observed that the concentration of formed CO for the molar ratio of CO_2_/H_2_O = 1:1 shows the peak soon after the start of illumination of Xe lamp and decreases gradually. It is also observed that the concentration of formed CO for the molar ratios of CO_2_/NH_3_/H_2_O = 1:1:1, 1:0.5:1, 3:2:3 are larger than that for the molar ratio of CO_2_/H_2_O = 1:1. In addition, the decrease of formed CO is small after the concentration of formed CO performs the highest value compared to the molar ratio of CO_2_/H_2_O = 1:1. Therefore, it is revealed that the combination of NH_3_ and H_2_O, that is, the existence of NH_3_ is effective for the promotion of the CO_2_ reduction performance of prepared photocatalyst. On the other hand, comparing the concentration change of CO along the time under the Xe lamp with UV light for the molar ratio of CO_2_/H_2_O = 1:0.5 to that for the molar ratio of CO_2_/NH_3_/H_2_O = 1:1:0.5, 1:0.5:0.5, it is observed that the concentration of formed CO for the molar ratio of CO_2_/H_2_O = 1:0.5 shows the peak soon after the start of illumination of Xe lamp and decreases gradually, which displays the same tendency as the result for the molar ratio of CO_2_/H_2_O = 1:1. It is also observed that the concentration of CO for the molar ratios of CO_2_/NH_3_/H_2_O = 1:1:0.5 and 1:0.5:0.5 are larger than that for the molar ratio of CO_2_/H_2_O = 1:0.5. In addition, the concentration of formed CO keeps some value approximately without rapid decrease before 24 h for CO_2_/NH_3_/H_2_O conditions compared to the molar ratio of CO_2_/H_2_O = 1:0.5. According to the reaction scheme to reduce CO_2_ with NH_3_ as shown above, the more reaction step is needed to produce CO since NH_3_ should be converted into H_2_ at first. Consequently, it is believed that the time to produce CO is longer compared to the molar ratio of CO_2_/H_2_O = 1:0.5. Moreover, comparing the concentration change of formed CO along the time under the Xe lamp with UV light for the molar ratio of CO_2_/H_2_O = 3:12 to that for the molar ratio of CO_2_/NH_3_/H_2_O = 3:8:12, it is observed that the concentration of formed CO for the molar ratio of CO_2_/H_2_O = 3:12 shows the peak soon after the illumination of Xe lamp and decreases gradually. In addition, the concentration of formed CO restarts to increase gradually and decrease again. This trend is different from the other CO_2_/H_2_O condition. The ratio of H_2_O is larger in this condition compared to the others, which indicates larger reductants provided for reduction reaction. Therefore, it is thought to keep CO production even though the oxidization reaction with CO and O_2_ starts, which is the reason for the decrease in concentration of CO. Furthermore, it is also observed that the concentration of formed CO for the molar ratio of CO_2_/NH_3_/H_2_O = 3:8:12 is larger than that for the molar ratio of CO_2_/H_2_O = 3:12. Consequently, it is revealed that the combination of NH_3_ and H_2_O, that is, the existence of NH_3_, is effective for promotion of the CO_2_ reduction performance of prepared photocatalyst for all conditions of CO_2_/NH_3_/H_2_O. When comparing the highest quantity produced CO in the case of CO_2_/NH_3_/H_2_O = 3:8:12 to that in the case of CO_2_/H_2_O = 3:12, it is confirmed that the highest produced CO in the case of molar ratio of CO_2_/NH_3_/H_2_O = 3:8:12 is approximately three times as large as that in the case of molar ratio of CO_2_/H_2_O = 3:12. Consequently, it is clear that NH_3_ could promote CO_2_ reduction performance of Cu/TiO_2_.

It can be seen from Table 2 that the CO_2_ reduction performance for the molar ratio of CO_2_/NH_3_/H_2_O = 1:0.5:0.5 is the highest where the molar quantity of CO per unit weight of photocatalyst is 2.5 μmol/g. In addition, Table 2 also reveals that the amount of total reductants required is smaller than that in the case with UV light shown in Table 1. When the Xe lamp is illuminated without UV light, the light intensity and wavelength range of light are smaller and narrower respectively, compared to the condition with UV light as described above. According to the reaction scheme of CO_2_ reduction with H_2_O or NH_3_ that an electron is produced by the photochemical reaction which is influenced by the light illumination condition. Additionally, H^+^ whose amount is the same as that of electron is needed to produce CO. Since the number produced electrons might be smaller due to the less light input without UV, it is believed that the numbers of required H^+^ are small. Therefore, the CO_2_ reduction performance for the molar ratio of CO_2_/NH_3_/H_2_O = 1:0.5:0.5 was the highest, while total reductants required were smaller than that in the case with UV light.

Table 3 and Table 4 show the trends in molar quantity of CO per unit weight of photocatalyst (Pd/TiO_2_) under the condition of Xe lamp illumination with and without UV light, respectively. Table 3 and Table 4 also list the maximum value of molar quantity of CO per unit weight of photocatalyst which is written in bold font. It is seen from Table 3 that the highest CO_2_ reduction performance reached at the illumination time of Xe lamp with UV light of 12 h irrespective of molar ratio of CO_2_/NH_3_/H_2_O. Though it is observed from Table 3 that the molar quantity of CO per unit weight of photocatalyst for the molar ratio of CO_2_/NH_3_/H_2_O = 3:2:3 has the highest value at the illumination time of 12 h, the molar quantity of CO per unit weight of photocatalyst decreased rapidly after 12 h. In these figures, the impact of molar ratio of CO_2_, NH_3_ and H_2_O is also presented. Additionally, the other fuels except for CO were not detected in this study. Before the experiment, a blank test without Xe lamp illumination had been carried out as a reference, resulting that no fuel was detected as expected. As to the reproducibility of experiments, this study shows the data from three averaged experiments.

According to Table 3, with the UV illumination, the CO_2_ reduction performance for the molar ratio of CO_2_/NH_3_/H_2_O = 1:1:1 is the highest where the molar quantity of CO per unit weight of photocatalyst is 5.5 μmol/g. Though this study assumes that the molar ratio of CO_2_/NH_3_/H_2_O = 3:2:3 is the theoretical molar ratios to produce CO, it is revealed that the optimum molar ratio of CO_2_/NH_3_/H_2_O is 1:1:1. Additionally, the molar ratio exhibiting the highest CO_2_ reduction performance for Pd/TiO_2_ is the same as that for Cu/TiO_2_. As described above, since the ionized Cu doped with TiO_2_ can provide free electron to be used for the reduction reaction process [39], the theoretically required quantity of the reductants of NH_3_ and H_2_O is reduced from the values according to the theoretical reaction scheme i.e., CO_2_/NH_3_/H_2_O = 3:3:3 to 3:2:3. According to the EELS spectra analysis as shown above, it is believed that Pd in Pd/TiO_2_ prepared in this study was in the form of Pd metal, which is the different from Cu^+^ ion in Cu/TiO_2_ prepared in this study.

Moreover, it can be seen from Table 4 that, without UV light illumination, the CO_2_ reduction performance for the molar ratio of CO_2_/NH_3_/H_2_O = 1:1:1 is the highest where the molar quantity of CO per unit weight of photocatalyst is 3.5 μmol/g. This optimum molar ratio (1:1:1) without UV light illumination is the same as the optimum molar ratio with UV light illumination. However, it is different from the optimum molar ratio obtained with Cu/TiO_2_ photocatalyst. Pd acts as an electron-transfer mediator, rapidly transferring the photoexcited electrons from the conduction band of Pd/TiO_2_ and the photoexcited electrons are transported to the surface of Pd/TiO_2_ [24]. Although Cu can also act an electron-transfer mediator, Pd might conduct the higher performance compared to Cu. As a result, Pd/TiO_2_ exhibits better CO_2_ reduction performance than Cu/TiO_2_ under the visible light illumination condition [24]. The amount of light absorbed by Pd might be enough even the smaller light input under the illumination condition of Xe lamp without UV light. As a result, the required amount of needed H^+^ for CO_2_ reduction without UV light is not smaller than the case with UV light, which is different from the case of Cu/TiO_2_ without UV light. Consequently, the CO_2_ reduction performance with Pd/TiO_2_ at the molar ratio of CO_2_/NH_3_/H_2_O = 1:1:1 is the highest for Pd/TiO_2_ even the illumination condition of Xe lamp without UV light.

### 3.3. The Quantum Efficiency Evaluation

Quantum efficiency is a well-known criterion used to indicate the photocatalytic activity and efficiency [41]. The quantum efficiency is generally calculated by the following equations [34,42]:

*η* = (*N*_output_/*N*_input_) ×100 (20)

*N*_input_ = (*I* × *t* × *λ* × *A*_re_)/(*h* × *c*) (21)

*N*_output_ = *N*_CO_*M*_CO_*N*_A_ (22)

where *η* is the quantum efficiency [%], *N*_input_ is the number of protons absorbed by photocatalyst [-], *N*_output_ is the photon number used in photocatalytic reaction [-], *I* is the light intensity of UV light [W/cm^2^], *t* is the time illuminating UV light [s], *λ* is the wave length limit of light to trigger the photocatalytic reaction by photocatalyst [m], *A*_re_ is the reaction surface area of photocatalyst assumed to be equal to the surface area of netlike glass disc [cm^2^], *h* is Plank’s constant (= 6.626 × 10^−34^) [J·s], *c* is light speed (= 2.998 × 10^9^) [m/s], *N*_co_ is the electron number required to form CO of a molecular (=2) [-], *M*_co_ is the molar number of formed CO [mol], *N*_A_ is Avogadro’s number (= 6.022 × 10^23^) [1/mol]. In this study, *I* averaged during all experiments where the illumination condition of Xe lamp with and without UV light were 58.7 mW/cm^2^ and 47.1 mW/cm^2^, respectively. *t* under both Xe lamp illumination condition with and without UV light were 345,600 s (96 h) in the case of Cu/TiO_2_ with UV light and without UV light as well as in the case of Pd/TiO_2_ without UV light, while *t* under both Xe lamp illumination condition with UV light was 43,200 s (12 h).

**Figure 9:**
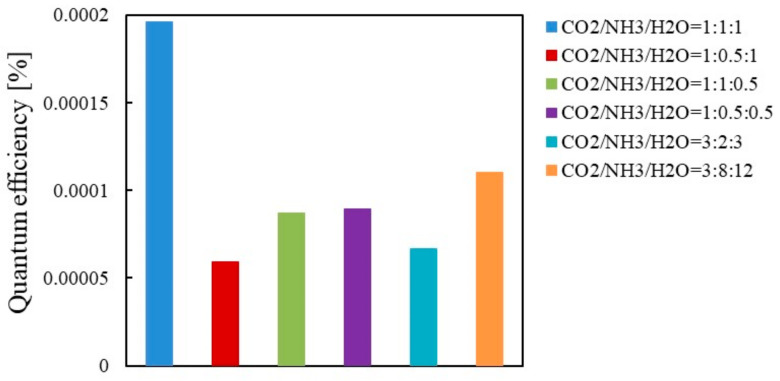
Comparison of quantum efficiency among different molar ratios for Cu/TiO_2_ under the illumination condition of Xe lamp with UV light.

**Figure 10:**
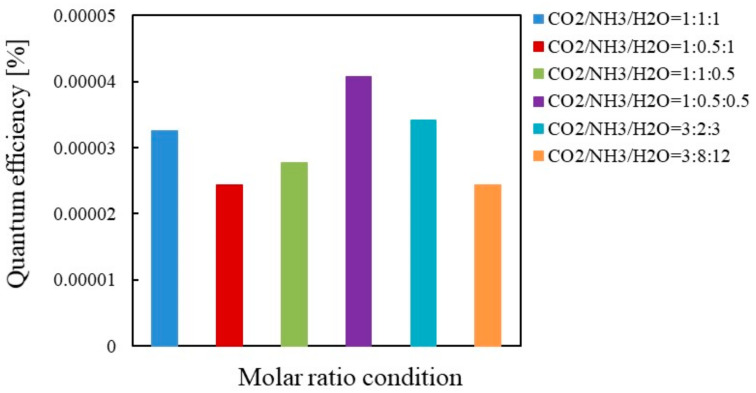
Comparison of quantum efficiency among different molar ratios for Cu/TiO_2_ under the illumination condition of Xe lamp without UV light.

Figure 9 and Figure 10 show the comparison of quantum efficiencies among different molar ratios of CO_2_/NH_3_/H_2_O for Cu/TiO_2_ under the condition of Xe lamp illumination with and without UV light, respectively. It is revealed from Figure 9 and Figure 10 that the highest quantum efficiency under the condition of Xe lamp illumination with and without UV light is obtained for the molar ratio of CO_2_/NH_3_/H_2_O = 1:1:1 and 1:0.5:0.5, respectively, which agrees with the results shown in Table 1 and Table 2. Comparing the quantum efficiencies shown in Figure 9 and Figure 10, the highest quantum efficiency of 1.96 × 10^−4^ is obtained when the Xe lamp with UV light is illuminated. If illumination time, *t* for the molar ratio of CO_2_/NH_3_/H_2_O = 1:1:1 with UV light is 6 h when the highest molar quantity of CO per unit weight of photocatalyst is obtained, the highest quantum efficiency for Cu/TiO_2_ is 3.14 × 10^−3^.

**Figure 11:**
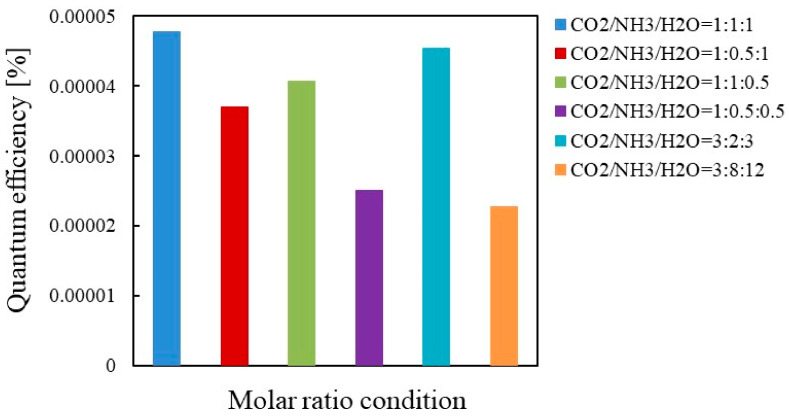
Comparison of quantum efficiency among different molar ratios for Pd/TiO_2_ under the illumination condition of Xe lamp with UV light.

**Figure 12:**
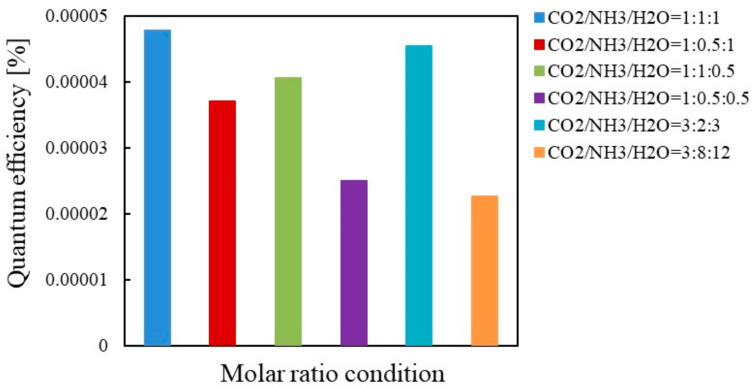
Comparison of quantum efficiency among different molar ratios for Pd/TiO_2_ under the illumination condition of Xe lamp without UV light.

Figure 11 and Figure 12 show the comparison of quantum efficiencies among different molar ratios of CO_2_/NH_3_/H_2_O for Pd/TiO_2_ under the condition of Xe lamp illumination with and without UV light, respectively. Figure 11 and Figure 12 reveal that the highest quantum efficiency under the condition of Xe lamp illumination with and without UV light is obtained when the molar ratio of CO_2_/NH_3_/H_2_O is 1:1:1, which agrees the results shown in Table 3 and Table 4. Comparing the quantum efficiencies shown in Figure 11 and Figure 12, the highest quantum efficiency of 4.20×10^−4^ is obtained with UV light. If *t* is set at 96 h which is the same time as the case of Cu/TiO_2_, the highest quantum efficiency for Pd/TiO_2_ under the condition of Xe lamp illumination with UV light is 0.53 × 10^−4^.

According to the previous study [39], Cu/TiO_2_ (2 wt% of Cu) photocatalyst performed the quantum efficiency of producing CO of 1.56 × 10^−2^ in the case of CO_2_/H_2_O with UV light. Another study reported that Cu/TiO_2_ (1 wt% of Cu) performed the quantum efficiency of 1.41 × 10^−2^ in the case of CO_2_/H_2_O with UV light [43]. As to Pd/TiO_2_, there is no previous study evaluating quantum efficiency of CO production, except for the report [26] which estimated the quantum efficiency of producing CH_4_ of 1.49 with Pd/TiO_2_ (1 wt% of Pd) and NaOH solution as the reductant.

The quantum efficiency obtained in the current study is lower than that obtained in previous studies. The reason is thought to be that the total amount of electron needed in this study for photochemical reaction is too large due to the combination of two H^+^ supplies i.e., NH_3_ and H_2_O. It is thought that, (i) capturing the maximum visible light region, and (ii) draining the photogenerated charges on light irradiation towards Cu/TiO_2_ surface [44], which may be possible ways to improve the quantum efficiency.

This study has confirmed that Cu/TiO_2_ is superior to Pd/TiO_2_ from the viewpoint of the molar quantity of CO per unit weight of photocatalyst as well as the quantum efficiency. As the next step, it can be considered to improve the CO_2_ reduction performance of TiO_2_ with NH_3_ and H_2_O further. Combination of different doped metals is one way to promote the CO_2_ reduction performance further in the near future. According to the previous studies [42,45,46], the co-doped TiO_2_ such as PbS-Cu/TiO_2_, Cu-Fe/TiO_2_, Cu-Ce/TiO_2_, Cu-Mn/TiO_2_, Cu-CdS/TiO_2_ and Au-Pd/TiO_2_ were able to promote the CO_2_ reduction performance of TiO_2_ with H_2_O. For the combination of CO_2_/NH_3_/H_2_O, the ion number of the dopant had better match the number of electrons emitted from the dopant with that of H^+^ according to the reaction scheme shown above. The same number of electrons and H^+^ is necessary to produce fuel from CO_2_. Although this study dopes only one metal in order to promote the CO_2_ reduction performance using TiO_2_, co-doping metals, which have larger positive ions compared to Cu, can provide a positive effect to promote the CO_2_ reduction performance with NH_3_ and H_2_O. Therefore, it is expected that the CO_2_ reduction performance will be promoted by the combination of different doped metals in the case of CO_2_/NH_3_/H_2_O.

## 4. Conclusions

From the investigation in this study, the following conclusions can be drawn:

(1) TiO_2_ film coated on netlike glass fiber was teeth like. Cu and Pd particles were loaded on TiO_2_ film uniformly. It is confirmed that the pulse arc plasma gun method can control the amount of metal doped on TiO_2_ irrespective of metal type.
(2) Cu in Cu/TiO_2_ prepared in this study exists as Cu^+^ ion in Cu_2_O. Pd in Pd/TiO_2_ prepared in this study exists as Pd metal.
(3) In the case of Cu/TiO_2_ under the illumination condition of Xe lamp with UV light, the CO_2_ reduction performance for the molar ratio of CO_2_/NH_3_/H_2_O = 1:1:1 was the highest where the molar quantity of CO per unit weight of photocatalyst was up to 10.2 mol/g.
(4) In the case of Cu/TiO_2_ under the illumination condition of Xe lamp without UV light, the CO_2_ reduction performance for the molar ratio of CO_2_/NH_3_/H_2_O = 1:0.5:0.5 was the highest where the molar quantity of CO per unit weight of photocatalyst was 2.5 mol/g.
(5) In the case of Pd/TiO_2_ under the illumination condition of Xe lamp with UV light, the CO_2_ reduction performance for the molar ratio of CO_2_/NH_3_/H_2_O = 1:1:1 was the highest where the molar quantity of CO per unit weight of photocatalyst was up to 5.5 mol/g.
(6) In the case of Pd/TiO_2_ under the illumination condition of Xe lamp without UV light, the CO_2_ reduction performance for the molar ratio of CO_2_/NH_3_/H_2_O = 1:1:1 was the highest where the molar quantity of CO per unit weight of photocatalyst was up to 3.5 mol/g.
(7) As to Cu/TiO_2_, the highest quantum efficiency was 1.96 × 10^−4^ under the illumination condition of Xe lamp with UV light. On the other hand, it was 3.14 × 10^−3^ if *t* was set at 6 h when the highest molar quantity of CO per unit weight of photocatalyst was obtained.
(8) As to Pd/TiO_2_, the highest quantum efficiency was 4.20 × 10^−4^ under the illumination condition of Xe lamp with UV light. On the other hand, it was 0.53 × 10^−4^ if *t* was set at 96 h which was the same illumination time of Xe lamp as Cu/TiO_2_.

## Figures and Tables

**Table 1:** Comparison of molar quantity of CO per unit weight of photocatalyst for Cu/TiO_2_ under the illumination condition of Xe lamp with UV light (unit: μmol/g).

Time [h]	0	3	6	9	12	15	18	21	24	48	72	96
CO_2_:NH_3_:H_2_O = 1:1:1	0	6.3	**10.2**	9.5	8.5	8.4	7.4	6.8	5.7	3.8	6.6	4.5
CO_2_:NH_3_:H_2_O = 1:0.5:1	0	3.0	4.4	5.1	5.0	4.8	**5.3**	5.0	5.0	3.1	2.6	2.7
CO_2_:NH_3_:H_2_O = 1:1:0.5	0	4.8	6.3	6.5	7.1	6.8	6.8	6.8	**7.9**	4.7	5.4	3.9
CO_2_:NH_3_:H_2_O = 1:0.5:0.5	0	5.3	7.0	5.0	6.4	7.7	**8.0**	6.5	5.1	3.8	3.6	4.2
CO_2_:NH_3_:H_2_O = 3:2:3	0	4.6	5.9	**6.0**	4.7	5.4	5.9	5.8	4.0	3.6	1.7	2.3
CO_2_:NH_3_:H_2_O = 3:8:12	0	3.4	5.7	6.3	**6.6**	4.7	4.3	4.6	5.1	3.5	4.8	6.2

**Table 2:** Comparison of molar quantity of CO per unit weight of photocatalyst for Cu/TiO_2_ under the illumination condition of Xe lamp without UV light (unit: μmol/g).

Time [h]	0	3	6	9	12	15	18	21	24	48	72	96
CO_2_:NH_3_:H_2_O = 1:1:1	0	0.6	0.8	0.9	0.9	1.0	1.2	1.4	2.0	**1.9**	1.5	1.2
CO_2_:NH_3_:H_2_O = 1:0.5:1	0	0.9	1.0	1.3	1.1	1.4	**1.6**	1.1	1.0	0.7	0.9	1.1
CO_2_:NH_3_:H_2_O = 1:1:0.5	0	0.8	1.4	**1.7**	1.6	1.4	1.2	1.1	1.0	1.3	1.0	1.6
CO_2_:NH_3_:H_2_O = 1:0.5:0.5	0	0.5	1.2	1.4	1.4	2.1	**2.5**	1.7	1.3	1.7	2.3	1.7
CO_2_:NH_3_:H_2_O = 3:2:3	0	0.5	1.1	1.6	1.4	0.9	1.3	1.5	**2.2**	1.4	1.1	1.3
CO_2_:NH_3_:H_2_O = 3:8:12	0	0.4	1.1	1.5	1.0	0.7	0.6	0.9	0.9	1.1	**1.5**	1.4

**Table 3:** Comparison of molar quantity of CO per unit weight of photocatalyst for Pd/TiO_2_ under the illumination condition of Xe lamp with UV light (unit: μmol/g).

Time [h]	0	3	6	9	12
CO_2_:NH_3_:H_2_O = 1:1:1	0	1.6	**5.5**	5.0	2.8
CO_2_:NH_3_:H_2_O = 1:0.5:1	0	1.4	**1.4**	0	0.1
CO_2_:NH_3_:H_2_O = 1:1:0.5	0	**2.1**	1.1	1.1	1.4
CO_2_:NH_3_:H_2_O = 1:0.5:0.5	0	**2.0**	1.5	0.8	1.2
CO_2_:NH_3_:H_2_O = 3:2:3	0	1.5	1.6	1.4	**2.4**
CO_2_:NH_3_:H_2_O = 3:8:12	0	1.7	1.2	**1.8**	1.1

**Table 4:** Comparison of molar quantity of CO per unit weight of photocatalyst for Pd/TiO_2_ under the illumination condition of Xe lamp without UV light (unit: μmol/g).

Time [h]	0	24	48	72	96
CO_2_:NH_3_:H_2_O = 1:1:1	0	1.7	**3.5**	3.2	3.1
CO_2_:NH_3_:H_2_O = 1:0.5:1	0	1.8	2.0	2.1	**2.5**
CO_2_:NH_3_:H_2_O = 1:1:0.5	0	2.5	2.3	**3.1**	1.6
CO_2_:NH_3_:H_2_O = 1:0.5:0.5	0	**2.5**	1.4	2.0	2.0
CO_2_:NH_3_:H_2_O = 3:2:3	0	1.6	1.6	**3.0**	2.8
CO_2_:NH_3_:H_2_O = 3:8:12	0	1.7	1.7	1.5	**1.8**

## Data Availability

The data presented in this study are openly available for all figures and tables.

## Sample Availability

Samples of the compounds are not available from the authors.
